# Construct validity of a questionnaire for measuring student engagement in problem-based learning tutorials

**DOI:** 10.1186/s12909-023-04820-1

**Published:** 2023-11-07

**Authors:** Salah Eldin Kassab, Amany El-Baz, Nahla Hassan, Hossam Hamdy, Silvia Mamede, Henk G. Schmidt

**Affiliations:** 1 College of Medicine, Gulf Medical University, Ajman, United Arab Emirates; 2 Department of Physiology, Faculty of Medicine, Suez Canal University, Ismailia, Egypt; 3 Department of Medical Education, Faculty of Medicine, Suez Canal University, Ismailia, Egypt; 4 Institute of Medical Education Research, Erasmus MC, Rotterdam, The Netherlands; 5 Department of Psychology, Education and Child Studies, Erasmus University Rotterdam, Rotterdam, The Netherlands

**Keywords:** Student engagement, Problem-based learning, Psychometrics, Validity, Questionnaire

## Abstract

**Background:**

Student engagement is student investment of time and energy in academic and non-academic experiences that include learning, teaching, research, governance, and community activities. Although previous studies provided some evidence of measuring student engagement in PBL tutorials, there are no existing quantitative studies in which cognitive, behavioral, and emotional engagement of students in PBL tutorials is measured. Therefore, this study aims to develop and examine the construct validity of a questionnaire for measuring cognitive, behavioral, and emotional engagement of students in PBL tutorials.

**Methods:**

A 15-item questionnaire was developed guided by a previously published conceptual framework of student engagement. Focus group discussion (*n* = 12) with medical education experts was then conducted and the questionnaire was piloted with medical students. The questionnaire was then distributed to year 2 and 3 medical students (*n* = 176) in problem-based tutorial groups at the end of an integrated course, where PBL is the main strategy of learning. The validity of the internal structure of the questionnaire was tested by confirmatory factor analysis using structural equation modeling assuming five different models. Predictive validity evidence of the questionnaire was studied by examining the correlations between students’ engagement and academic achievement.

**Results:**

Confirmatory factor analysis indicates a good fit between the measurement and structural model of an 11-item questionnaire composed of a three-factor structure: behavioral engagement (3 items), emotional engagement (4 items), and cognitive engagement (4 items). Models in which the three latent factors were considered semi-independent provided the best fit. The construct reliabilities of behavioral, cognitive, and emotional factors were 0.82, 0.82, and 0.76, respectively. We failed however to find significant relationships between academic achievement and engagement.

**Conclusions:**

We found a strong evidence to support the construct validity of a three-factor structure of student engagement in PBL tutorial questionnaire. Further studies are required to test the validity of this instrument in other educational settings. The predictive validity is another area needing further scrutiny.

**Supplementary Information:**

The online version contains supplementary material available at 10.1186/s12909-023-04820-1.

## Introduction

Studies have demonstrated that student engagement is one of the most robust predictors of academic achievement [1] and increased student perseverance and retention. In addition, student engagement correlates with desirable mental health outcomes such as low rates of depression [2], and higher life satisfaction [3]. Student engagement is also intrinsically rewarding for teachers [4], while student disengagement is a major factor for teacher burnout [5]. Furthermore, student engagement is recognized as a measure of institutional quality [6] and excellence [7]. Despite the escalating interest in the construct of student engagement, there are several gaps in the medical education literature about its measurement and applications in different educational settings.

Recently, we conducted a scoping review on student engagement in undergraduate medical education [8] and developed an integrated conceptual framework for student engagement in health professions education [9]. This comprehensive framework contained the antecedents, mediators, dimensions, spheres, and outcomes of student engagement. According to this framework, student engagement comprises five dimensions: cognitive, behavioral, emotional, agentic, and socio-cultural. Cognitive engagement involves the student's psychological investment in learning, going beyond mere requirements, seeking challenges, directing effort towards understanding and mastering content, and utilizing metacognitive and deep learning strategies [10]. Behavioral engagement refers to positive conduct, persistence, directing effort towards completing learning tasks, active participation, asking questions, maintaining focus, and engaging in school-based activities. Emotional engagement pertains to the student's emotional reactions in the classroom, school, or towards teachers, such as experiencing enjoyment, interest, happiness, and a sense of bonding [10]. Agentic engagement emphasizes the active role of students in shaping their educational paths, future social lives, and broader social environments [11]. Indicators of agentic engagement within the classroom may manifest as students actively contributing to their learning and influencing the instructional process [12]. Agentic engagement beyond the classroom might encompass students taking an active role in community initiatives, engaging in peer teaching and mentoring, as well as participating in institutional governance and quality assurance efforts [9]. Finally, socio-cultural engagement refers to the students’ quality of interactions with, accepting to learn from, and predicting actions of, different social and cultural groups [13]. Sociocultural engagement develops when students immerse themselves in a new social setting and develop their unique identities. This process of identity formation helps bridge the gap between their personal social and cultural values and the norms of the new community. Consequently, students may develop their identities by fostering a sense of belonging within the new community [14, 15]. In this article, we will confine ourselves to the study of the dimensions that are deemed directly important in the classroom setting: cognitive, emotional, and behavioral engagement. A second restriction is that we have focused on the problem-based small-group tutorial setting rather than on instructional environments in a broader sense. The study was conducted in such a setting.

Student engagement in problem-based learning (PBL) has been examined in previous studies [16–20]. Assessment of medical students’ engagement with direct observation demonstrated that the amount of learner-to-learner engagement was similar in PBL and team-based learning (TBL) [17, 18], and much greater than in lecture-based teaching [17, 19], where most engagement was of the learner-to-instructor and self-engagement types. Also, learner-to-instructor engagement appeared greater in TBL compared with PBL [17, 18]. Another study developed and validated a 4-item questionnaire for measuring situational cognitive engagement in the PBL classroom [20]. A recent study used video-stimulated recall as prompts for personal interviews to explore the dynamics of their engagement in PBL tutorial groups [21]. They demonstrated that engagement of students in one dimension leads to further engagement in other dimensions and that engagement is decided by students before the PBL session based on the available antecedents [21]. Although these studies have used different methods of measuring student engagement in PBL tutorials, the scope of quantitative measurement by the instruments has been limited to one dimension of engagement, either behavioral or cognitive. Accordingly, there are no existing studies in the literature which measure multiple dimensions of students’ engagement in PBL tutorials. Therefore, this study is designed to address the following research questions: 1. What is the content-related validity evidence of the questionnaire for measuring student engagement in PBL tutorials? 2. What is the internal structure validity evidence of the questionnaire for measuring student engagement in PBL tutorials? And 3. What is the relationship between student engagement in PBL tutorials and their academic achievement?

## Methods

### Overview

The present study used a cross-sectional correlation design. The questionnaire was designed based on a psychological perspective of engagement and the student engagement construct was considered multidimensional. Student engagement is conceptualized in this study as the student investment of time and energy in PBL tutorial experiences at the cognitive, affective, behavioral dimensions. An initial questionnaire was designed to operationalize the three dimensions of the student engagement construct based on our previously published conceptual framework of engagement [9]. A focus group discussion was then conducted with medical education experts (*n* = 12) who examined the degree of concordance between each item of the questionnaire and the intended construct and for examining the degree of clarity of the items. The outcome of the focus group discussion was that experts agreed to include all the 15 items with slight modifications and degrees of agreement ranging from 60 to 100% for items. The questionnaire was then pilot tested with a small group of year 2 medical students (*n* = 10) for suitability of the items and no further modifications were included.

### Setting and participants

The target population in this study were medical students in phase II of the medical program at a college of medicine in the Gulf region. The medical MBBS program at this college consists of five years duration. Year 1 (Phase I) is a foundation year with emphasis on basic medical sciences and general education courses. Year 2 and 3 (Phase II) consists of integrated medical sciences courses arranged in body systems. Problem based learning (PBL) is the main strategy of learning in Phase II of the program and PBL tutorials are the backbone activity. Year 4 and 5 (Phase III) consists of hospital-based rotations in different core clinical specialties.

The context of the study was the PBL small group tutorials conducted during an integrated system-based course. Small-group PBL tutorials consist of 8 to 10 students who meet twice a week for two hours in each session. The tutorials are led by a PBL tutor who functions mainly as a facilitator of learning rather than providing information. In the first session, students discuss a clinical case which is designed to stimulate rich discussion in the group and students generate a list of learning needs by the end of the session. Students then go into a stage of self-study scaffolded by structured college teaching activities between the first and second session. Students then meet again to present their learning during the week and integrate the information related to the case. Each PBL tutor is assigned to the group throughout the whole semester.

### Instruments and sampling

The final form of the study questionnaire consists of 15 items representing emotional engagement (4 items), cognitive engagement (6 items), and behavioral engagement (5 items). The multiple-choice achievement test consisted of 100 items of the A-type (single best response) and covered all contents of the course. Most of the questions are context-rich scenarios which test the application of knowledge rather than simple recall. We used convenient sampling with a targeted population size of 204 year 2 and 3 medical students. The paper-based questionnaire was filled in by 176 students (Response rate = 86%) at the end of an organ-system course. Students were informed to score their overall engagement in PBL tutorials during the course which ranged from 6 to 7 weeks.

### Statistical analysis

The purpose of the study was to collect different lines of evidence supporting the validity of the questionnaire. The data were entered and analyzed using the Statistical Package for Social Sciences (*SPSS*) version 25.0 and Analysis of Moment Structures (Amos) version 25.0 (Chicago, IBM SPSS). A *P*-value < 0.05 was considered statistically significant.

#### Confirmatory factor analysis

Confirmatory factor analysis using maximum likelihood estimation was applied to examine the degree of fit between the measurement model (the observed indicators) and the underlying structural model (the latent factors). Different indices were used to assess the goodness-of-fitness of the model. The Comparative Fit Index (CFI) assesses the overall performance of the model studied over a baseline (independence) model. Conventionally, CFI should be equal to or greater than 0.90 to accept the model. This denotes that 90% of the covariation in the data can be reproduced by the given model. The *Chi-Square* (χ^2^) test indicates the degree of fit between implied and observed covariance matrices. An insignificant χ^2^or a χ^2^ / df < 2 indicates good fit for the model. The Root Mean Square Error of Approximation (RMSEA) indicates the mean difference between observed and predicted covariance, and a value of 0.08 or less indicates an acceptable model fit. The Standardized Root Mean Square Residual (SRMR) is defined as the mean standardized difference between the observed correlation matrix and the model implied correlation matrix. A value less than 0.08 is considered a good fit. This measure tends to be smaller as sample size increases and as the number of parameters in the model increases [22]. Finally, often the Aikaike Information Criterion (AIC) is computed. The AIC compares all different possible models in terms of appropriate use of all information in the data. Lower AIC values indicate a better fit. In conclusion, a decision on what the best model fit represents always takes these different indicators into account.

We first test the full 15-item questionnaire data as envisioned by the focus groups of students and experts against the three-factor model. The results are: χ^2^ = 321.35, df = 90, χ^2^/df = 3.57, CFI = 0.85, RMSEA = 0.12, SRMR = 0.26, and AIC = 381.35. This model clearly did not fit the data. A possible reason was that four items had small loadings or did load on more than one factor. The item “*I feel the time passes quickly during the PBL tutorial*” cross-loaded with high regression weights on both cognitive and emotional engagement. The items “*I challenge myself in understanding the topics related to PBL case*” and” I *pay full attention to during the PBL tutorial*” loaded on both cognitive and behavioral engagement. On the other hand, the item “*I feel bored in PBL tutorials*” cross loaded on the three engagement dimensions. We therefore decided to continue the analysis with the remaining 11 items.

The first model assumed that all these Items loaded on the same engagement factor, suggesting that one latent factor was sufficient to explain the data. This model is the simplest possible and therefore in theory the most parsimonious. It does, however, not fit with the original theoretical analyses. The second model hypothesized three independent, latent factors. The assumption here is that indeed three latent factors, emotional, cognitive, and behavioral, would explain the data, but that these factors were uncorrelated. The third model assumed that the three factors were uncorrelated. However, since the data were acquired using the same method, it was hypothesized that the data shared common-method variance in the form of a fourth latent factor related to all items. This approach assumes that participants have a biased tendency to respond to all items in a somewhat similar way. Finally, the fourth model allowed the three latent factors to be correlated, assuming that emotion, cognition, and behaviors are (at least) to some extent in harmony.

#### Construct reliability (CR)

Composite (or construct) reliability is a measure of internal consistency in the observed indicators that load on a latent variable (construct). In structural equation modeling, the formula for calculating construct reliability is:

$$CR=\frac{{\left(\sum {\lambda }_{i}\right)}^{2}}{{\left(\sum {\lambda }_{i}\right)}^{2}+\left(\sum {\epsilon }_{i}\right)}$$

whereby, λ (lambda) is the standardized factor loading for item *i* and ε is the respective error variance for item *i*. The error variance (ε) is estimated based on the value of the standardized loading (λ) and appears in the Amos output [23].

#### Correlations with academic achievement

Correlations were computed between the three student engagement factors and their examination scores.

## Results

### Confirmatory factor analysis

The assessment of normality indicated a within acceptable range of skewness (between -.02 to 3.2) and kurtosis (-0.3 to -1.9). In Table 1, the most important findings are displayed.

**Table 1:** (Model fit for four measurement models of student engagement in PBL tutorials questionnaire (n= 176)

Model	χ^2^	df	χ^2^/ df	CFI	RMSEA (90% C.I.)	SRMR	AIC
1. One latent factor	585.82	44	13.31	.55	.27 (.25-.29)	.20	629.82
2. Three independent latent factors	158.11	44	3.59	.91	.12 (.10-.14)	.24	202.11
3. Three independent latent factors sharing common method variance (expressed as a fourth latent factor explaining all measured variables)	58.26	33	1.77	.98	.06 (.04-.08)	.04	124.26
4. Three latent factors allowed to correlate freely	82.52	41	1.88	.96	.07 (.05-.10)	.06	132.52

**Fig. 1:**
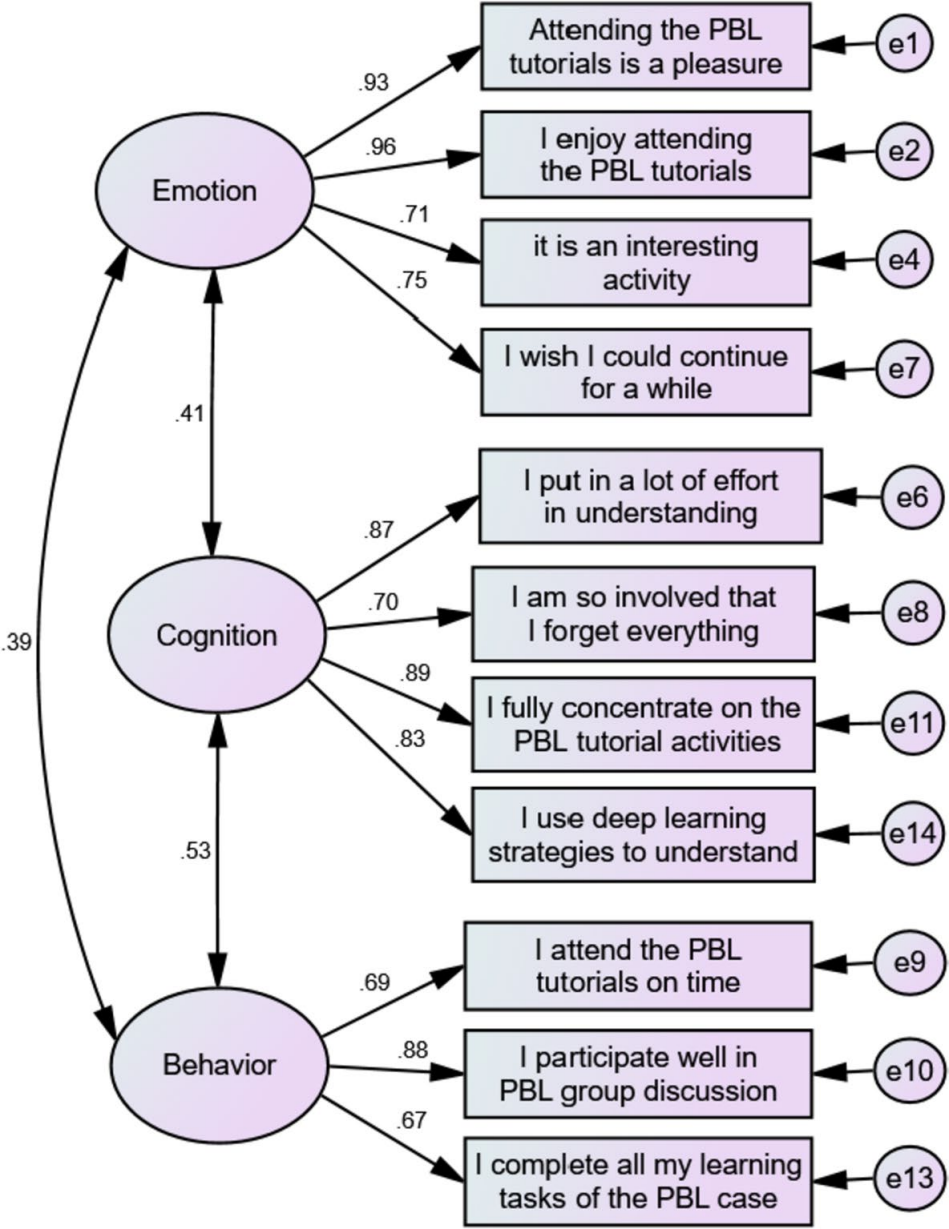
(Confirmatory factor analysis of medical student engagement in PBL tutorials questionnaire demonstrating the three-factor model with the latent factors correlated)

Scrutinizing the various fit indicators, both Model 3 and Model 4 fit the data well. Since Model 4 is the simplest one, and therefore the most parsimonious, we conducted further analyses with the latter. Figure 1 shows factor loadings for each of the items on the three latent factors.

All items loaded significantly on their respective factors, with standardized factor loadings ranging from .67 to .96, with the enjoyment, concentration, and participation items as the highest loading items.

### Construct reliability

Construct reliability was calculated for each factor. Construct reliability for cognitive engagement was .75, emotional engagement .80, and behavioral engagement .80.

### Correlations with academic achievement

There was a weak positive correlation between behavioral engagement and academic scores (*r* = .20). However, the relationships between emotional or cognitive engagement on the one hand and academic achievement on the other were not significant.

## Discussion

The purpose of the present study was to test reliability and construct validity of a multidimensional questionnaire measuring engagement of students in problem-based small-group tutorials. The results of the confirmatory factor analysis provided support for the reliability and validity of the 11-item questionnaire. The three-factor structure of the student engagement questionnaire (emotional, cognitive, and behavioral engagement) seems to be useful in guiding future research about student engagement in PBL tutorials. The predictive validity of the instrument, expressed as correlations between the three dimensions and academic achievement was limited to behavioral engagement.

Cognitive engagement of students in PBL tutorials entails their commitment to the process of learning, extending beyond the mere fulfillment of academic requirements. This includes dedicating effort towards comprehending and mastering the subject matter, immersion in the PBL tutorial that they lose awareness of the surroundings, completely focusing on the PBL tutorial tasks, and using deep strategies for their learning. Behavioral engagement in PBL tutorials involves actively taking part in group interactions, fulfilling all the learning activities associated with the PBL case, and punctually attending all tutorial sessions. Emotional engagement relates to the student’s affective responses during PBL tutorials, including feelings of enjoyment, interest, pleasure, and a desire for the tutorial to prolong.

Two previous studies examined the dynamics of student engagement in small group PBL tutorials [20, 21]. A study by Rotgans and Schmidt [20] was based on cognitive constructivism theory. Based on this perspective, they made three assumptions. First, students engage in *theory construction* during their encounter with a PBL case and test the theory through self-directed learning. Second, students develop an *interest*, ‘hunger for knowledge,’ when they encounter authentic cases. Third, students feel autonomy *and agency* through generating their own learning goals. Our findings support the study by Rotgans and Schmidt in that some of the items in the current instrument match with the construct of situational cognitive engagement [20]. We refer to the student’s feeling of being absorbed in the task and the fact that they put a lot of effort into working with the PBL case. However, the present instrument broadens the cognitive engagement construct to include concentration on the task and the use of deep learning strategies. In addition, the emotional and behavioral engagement constructs had no role in their approach.

A second study used a qualitative approach by conducting video-stimulated recall interviews to examine the dynamics of student engagement in PBL tutorials [21]. This study was explicitly guided by the multidimensional model of student engagement. The investigators found that student engagement should be described as a dynamic and malleable construct by which student engagement followed a spiral-like pattern. Once students were engaged or disengaged on one-dimension, other dimensions were likely to follow suit. Engagement is however a malleable variable. The degree of student engagement in PBL tutorials can change at any moment in a session depending on group dynamics, authenticity of the case scenario, or tutoring skills of faculty [20]. Therefore, it is suggested that methods of measurement should be selected to have the appropriate grain size, sensitive to pick up the contextual changes in student engagement at the level of PBL tutorial activity. To be able to measure these contextual changes, measures like direct observation and experience sampling using short questionnaires should be used. This represents a difficult tradeoff between measuring the multidimensionality of the construct or identifying the short-term changes in student engagement during the PBL tutorial activity. The questionnaire tested here represents a compromise that can be used to measure short-term changes in student engagement. Although the study was conducted to measure student engagement in PBL tutorials at the course level, this 11-item questionnaire requires around 25 s to fill in, which makes it suitable for identifying the dynamic and temporal changes in student engagement at the microlevel.

An analysis of the items that were removed from the original instrument provides insights into the dynamics of the constructs themselves. The item “*I feel the time passes quickly during the PBL tutorial*” loaded high on both cognitive and emotional engagement constructs. When students are emotionally engaged, they enjoy the learning experience and may feel a sense of flow, in which they are fully absorbed in the learning experience and lose track of time. At the same time, if a learner is cognitively engaged, they are actively processing information, making connections, and make them lose time awareness as well. The item “*I challenge myself in understanding the topics related to PBL case*” loaded on both cognitive and behavioral engagement. Although the act of challenging oneself is primarily a cognitive engagement process, the challenge to understand a topic may lead to increased behavioral engagement such as increased participation in class or completion of assignments. Similarly, the item” I *pay full attention to during the PBL tutorial*” loaded on both cognitive and behavioral engagement. When a student pays attention in class, they are actively engaging their cognitive processes to process and understand the information being presented. On the other hand, when a student pays attention in class, they are demonstrating behavioral engagement by actively participating in the learning process and demonstrating a willingness to learn. Therefore, paying attention in class involves both cognitive and behavioral engagement, as it involves both mental and observable behaviors that reflect a student’s engagement in the learning process.

## Limitations

Somewhat disappointedly, the predictive validity of the questionnaire fell short to some extent. A correlation of *r* = .20 between behavioral engagement and academic achievement was found, whereas the two other elements were not correlated with achievement. Another study found a similarly low correlation between engagement and achievement in the context of small-group education [24]. We can here only speculate about the reasons for this finding. A well-known phenomenon in forms of education stressing student agency and autonomy in pursuing learning issues is that the achievement test, representing what the teacher saw as the objectives of the course, does only fit what students actually study to some extent. Dolmans and colleagues [25] demonstrated the topics that students spent time and energy on in a problem-based course matched on average 64% with faculty-generated topics. In another study, they were able to show that student-generated learning issues, not foreseen by the teacher, often nevertheless were relevant for the problems at hand [26]. This discrepancy may be a reason that correlations between engagement and achievement in these contexts remain low. Another possible explanation is the lack of full alignment between the expected outcomes of PBL tutorials and the achievement test. Multiple choice questions are mainly designed to assess the cognitive dimension of learning. However, the learning outcomes of PBL tutorials include other competencies which cannot be measured using MCQs such as communication skills, interpersonal skills, problem solving, and self-directed learning.

### Supplementary Information

**Additional file 1. **

## Data Availability

The datasets used and/or analyzed during the current study are available from the corresponding author on reasonable request.
