# Supplementary material for: Construct validity of a questionnaire for measuring student engagement in problem-based learning tutorials
Source: BMC Med Educ. 2023 Nov 7;23:844. doi: 10.1186/s12909-023-04820-1 (PMC10631064; doi:10.1186/s12909-023-04820-1)
Supplement: Supplementary file 1 — Additional file 1. [file 12909_2023_4820_MOESM1_ESM.docx]

**Medical students engagement in PBL tutorials questionnaire**

**Dear student:**

Listed below are statements describing different aspects of your engagement in the PBL tutorial during this course. In your judgment, for each of the following statements, please indicate how true it is for you, using the following scale? (Circle your answer according to the scale below)

| **1** | **2** | **3** | **4** | **5** | **6** | **7** |
| --- | --- | --- | --- | --- | --- | --- |
| **Very untrue of me** | Untrue of me | Somewhat untrue of me | **Neutral** | Somewhat true of me | True of me | **Very**  **true of me** |

|  |  | Very  untrue of me |  |  |  |  |  | Very  true of me |
| --- | --- | --- | --- | --- | --- | --- | --- | --- |
| 1. | Attending the PBL tutorials is a pleasure | 1 | 2 | 3 | 4 | 5 | 6 | 7 |
| 2. | I enjoy attending the PBL tutorials | 1 | 2 | 3 | 4 | 5 | 6 | 7 |
| 3. | I enjoy the PBL tutorial because it is an interesting activity | 1 | 2 | 3 | 4 | 5 | 6 | 7 |
| 4. | I put in a lot of effort in understanding the topics of the PBL case | 1 | 2 | 3 | 4 | 5 | 6 | 7 |
| 5. | I wish I could continue for a while in this PBL tutorials | 1 | 2 | 3 | 4 | 5 | 6 | 7 |
| 6. | I am so involved that I forget everything around me in the PBL tutorials | 1 | 2 | 3 | 4 | 5 | 6 | 7 |
| 7. | I attend the PBL tutorials on time | 1 | 2 | 3 | 4 | 5 | 6 | 7 |
| 8. | I participate well in PBL group discussion | 1 | 2 | 3 | 4 | 5 | 6 | 7 |
| 9. | I fully concentrate on the PBL tutorial activities | 1 | 2 | 3 | 4 | 5 | 6 | 7 |
| 10. | I complete all my learning tasks of the PBL case | 1 | 2 | 3 | 4 | 5 | 6 | 7 |
| 11. | I use deep learning strategies to understand the content of the PBL case | 1 | 2 | 3 | 4 | 5 | 6 | 7 |
